# How and When Should NSAIDs Be Used for Preventing Post-ERCP Pancreatitis? A Systematic Review and Meta-Analysis

**DOI:** 10.1371/journal.pone.0092922

**Published:** 2014-03-27

**Authors:** Ignasi Puig, Xavier Calvet, Mireia Baylina, Álvaro Isava, Pau Sort, Jordina Llaó, Francesc Porta, Francesc Vida

**Affiliations:** 1 Digestive Diseases Department, Althaia Xarxa Assistencial Universitària de Manresa, Barcelona, Spain; 2 Universitat Internacional de Catalunya, Barcelona, Spain; 3 Departament de Medicina, Universitat Autònoma de Barcelona, Barcelona, Spain; 4 Digestive Diseases Department, Corporació Sanitària Parc Taulí, Sabadell, Barcelona, Spain; 5 Centro de Investigación Biomédica en Red de enfermedades hepáticas y digestivas (CIBERehd), Madrid, Spain; 6 Internal Medicine Department, Corporació Sanitària Parc Taulí, Sabadell, Barcelona, Spain; University of Valencia, Spain

## Abstract

**Background:**

Non-steroidal anti-inflammatory drugs (NSAIDs) have been shown to be efficacious to prevent pancreatitis after endoscopic retrograde cholangiopancreatography (ERCP). However, the target patients, the type of NSAID, the route of administration and the time of drug delivery remain unclear, as well as the potential efficacy in reducing the severity of pancreatitis, length of hospital stay and mortality. The objective of the study was to evaluate these questions by performing a systematic review and meta-analysis.

**Methods:**

Multiple searches were performed in the main databases. Randomized controlled trials (RCTs) comparing NSAIDs vs. placebo in the prevention of post-ERCP pancreatitis were included. Primary endpoint of the study was the efficacy for pancreatitis prevention. Sub-analyses were performed to determine the risk reduction in high and low risk patients, and to define optimal time, route of administration, and type of NSAID. Secondary endpoints were safety, moderate to severe pancreatitis prevention and reduction of hospital stay and mortality.

**Results:**

Nine RCTs enrolling 2133 patients were included. The risk of pancreatitis was lower in the NSAID group than in the placebo group (RR 0.51; 95%CI 0.39–0.66). The number needed to treat was 14. The risk of moderate to severe pancreatitis was also lower in the NSAID group. (RR 0.46; 95%CI 0.28–0.76). No adverse events related to NSAID use were reported. NSAIDs were effective in both high-risk and unselected patients (RR 0.53; 95%CI 0.30–0.93 and RR 0.57; 95%CI 0.37–0.88). In the subanalyses, only rectal administration of either indomethacin (RR 0.54; 95%CI 0.38–0.75) or diclofenac (RR 0.42; 95%CI 0.21–0.84) was shown to be effective. There were not enough data to perform a meta-analysis in hospital stay reduction. No deaths occurred.

**Conclusion:**

A single rectal dose of indomethacin or diclofenac before or immediately after ERCP is safe and prevents procedure-related pancreatitis both in high risk and in unselected patients.

## Introduction

Endoscopic retrograde cholangiopancreatography (ERCP) is a widely used procedure that combines upper gastrointestinal endoscopy and radiography to diagnose and treat bile- and pancreas-related diseases such as choledocholithiasis, benign and malignant strictures, and so on. It is estimated that 500,000 procedures are performed annually in the United States [1]. The most common complication of ERCP is pancreatitis, occurring in 2–9% of patients in unselected prospective series [1]. It is associated with substantial morbidity and long hospitalization, although mortality is rare [2], [3]. Diagnostic criteria for post-ERCP pancreatitis (PEP) are new onset of pancreatic-type abdominal pain and amylase or lipase at least three times the normal rate more than 24 hours after the procedure requiring hospital admission or a prolongation of planned admission [4].

It is widely accepted that the local and systemic inflammatory response induced by ERCP is the physiopathological event that triggers PEP [5]–[7]. It has been proposed that phospholipase A2 (PLA2) plays an important role in the pathogenesis of this inflammatory response [5]. In vitro assays show that non-steroidal anti-inflammatory drugs (NSAIDs) are potent inhibitors of PLA2 activity in the serum in patients with severe acute pancreatitis and indomethacin and diclofenac are the most effective PLA2 inhibitors [8].

The fact that the initial triggering event of PEP is well defined has prompted researchers to seek out measures for its prevention. Pancreatic stent is not performed by all endoscopists because stent insertion may be difficult in patients with small or tortuous ducts, and there is a risk of pancreatic ductal injury [9]. Furthermore, a follow-up endoscopy is necessary for stent removal. For all these reasons, this procedure is not widely applied and an effective protective pharmacological agent would be of great benefit. The results of RCTs using nitroglycerine, ceftazidime, somatostatin, octreotide, antiprotease drugs, glucocorticoids, drugs reducing sphincter of Oddi pressure, antioxidant drugs, heparin, and Interleukin-10 have been disappointing [4]. Some studies have shown a benefit with NSAIDs [10]–[18], but a practice survey study performed some years ago showed that they were not widely used [19]. The main reason quoted was insufficient supporting evidence, but the authors speculate that clinicians’ scepticism related to the failure of many other large studies with other pharmacological agents also played an important role.

Previous meta-analyses have suggested that NSAIDs are effective in preventing post-ERCP pancreatitis [20]–[22], and the ESGE guidelines [4], based on four RCTs, recommend routine rectal administration of 100 mg of diclofenac or indomethacin immediately before or after ERCP. For this reason, NSAID use is increasing rapidly. However, after the publication of more recent studies, certain clinically relevant issues regarding the drug administration and the target patients remain unresolved. The rectal route is uncomfortable and drugs’ absorption may be erratic. For this reason, it may be useful to determine whether the parenteral route is also effective. In addition, whether NSAIDs should be given only to selected patients at high risk of developing PEP, or to all patient who undergo ERCP, is still a matter of debate. We conducted a systematic review and meta-analysis in order to determine the effectiveness of non-rectal and rectal NSAIDs, to establish the optimal moment of drug delivery and the most appropriate type of NSAID, and to assess their efficacy according to risk factors for developing pancreatitis. We also evaluated their safety and the efficacy for reducing pancreatitis severity, length of hospital stay, and mortality.

## Methods

**Figure 1:**
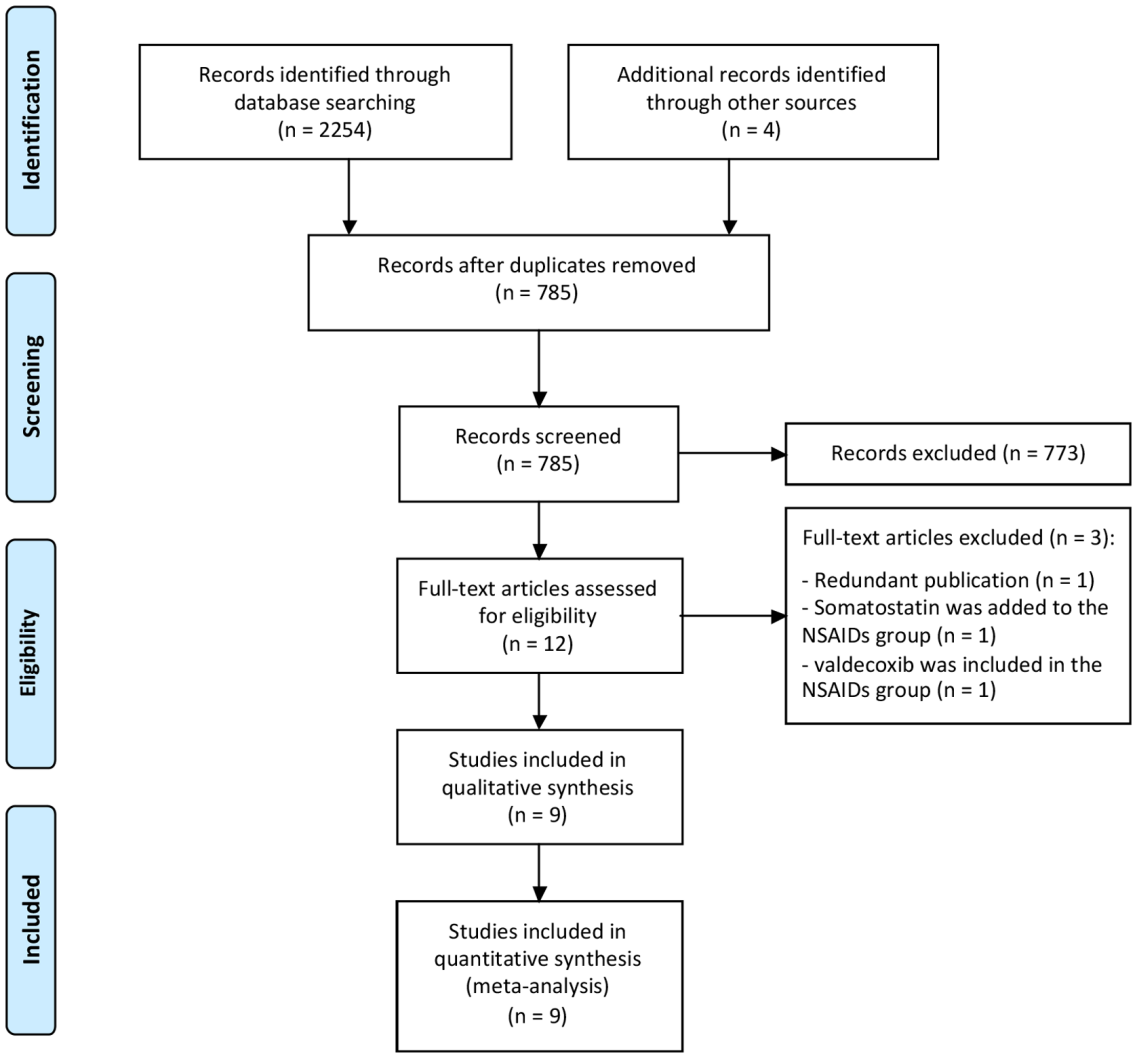
Flow diagram of included and excluded trials.

The study was performed in accordance with the PRISMA recommendations for systematic reviews and meta-analyses [23]. The PRISMA 2009 checklist is shown in Checklist S1 and the PRISMA 2009 flow diagram in Figure 1. We did not register the protocol.

### Search strategy

Multiple searches were performed in Scopus, Medline, the Cochrane Library database and the ISI Web of Knowledge from 1980 to November 2013. Details of the search are shown in Appendix S1. In addition, the references of the selected articles and those of significant reviews on the topic were also examined for articles missed in the previous searches.

### Inclusion criteria

Published studies were included if: a) they evaluated the efficacy of NSAIDs for prevention of post-ERCP pancreatitis; b) they were randomized controlled trials with a placebo arm; 3) they reported the incidence of post-ERCP pancreatitis in each arm.

### Definitions

Post-ERCP pancreatitis (PEP) was defined according to previous consensus as: ”clinical pancreatitis with amylase at least three times the normal rate more than 24 hours after the procedure, requiring hospital admission or a prolongation of planned admission” [24]. However, subsequent modified definitions that specified the meaning of “clinical pancreatitis” were also accepted [4]: “new or worsened abdominal pain” [25], “typical pain and symptoms” [26] or “abdominal pain and tenderness” [27].

Risk factors for developing PEP were extracted from previous reviews and meta-analyses [4], [28]: female sex, young age, suspected SOH, prior post-ERCP pancreatitis, recurrent pancreatitis, pancreatic duct injection, pancreatic sphincterotomy, balloon dilatation, difficult or failed cannulation, precut sphincterotomy and ampullectomy.

Regarding the inclusion criteria, a study was considered to involve high-risk patients when all patients included presented at least one risk factor. On the other hand, a study was considered to include unselected patients if the presence of risk factors was not a criterion for inclusion in the study. In this case, studies could include either all patients who underwent ERCP or patients with suspected bile obstruction, which is not considered an independent risk factor for PEP.

### Endpoints of the study

The primary endpoint of the study was the efficacy of NSAIDs for preventing PEP. This was evaluated by comparing the number of patients presenting PEP in the NSAIDs group *vs.* the placebo group.

Sub-analyses of the primary endpoint were planned to compare the incidence of PEP according to: a) the type of NSAID (indomethacin or diclofenac); b) route of administration (rectal or non-rectal); c) time of administration (before or after ERCP); d) risk of pancreatitis according to inclusion criteria (high risk or unselected patients); e) presence of each individual risk factor (female sex, young age, suspected SOH, prior post-ERCP pancreatitis, recurrent pancreatitis, pancreatic duct injection, pancreatic sphincterotomy, balloon dilatation, difficult or failed cannulation, pre-cut sphincterotomy and ampullectomy); f) the placement of a pancreatic stent.

Most subanalyses were performed separating the studies in the subgroup in question according to study design, but the two last subanalyses (each individual risk factor and the placement of a pancreatic stent) were based on the data extracted from the stratified results of each study (i.e. the number of PEP in females receiving NSAIDs *vs.* the number of PEP in females receiving placebo). Studies reporting only the baseline characteristics of patients (not the incidence of PEP in each subgroup) were not included.

Secondary endpoints were the efficacy of NSAIDs for reducing the number of moderate and severe pancreatitis, number of adverse events related to NSAIDs, length of hospital stay, and mortality. They were summarized through a quantitative meta-analysis when feasible, or described otherwise.

### Data extraction

Data extraction was performed in duplicate by two observers (MB & AI). Discordances were resolved by consensus with a third observer (IP). For each study we extracted the following variables: first author, year of publication, country, inclusion criteria, exclusion criteria, the definition of pancreatitis, differences in baseline risk factors between the groups, the use of pancreatic stent for PEP prophylaxis, the type, schedule and route of administration of the NSAID, the incidence of PEP in all groups, the incidence of PEP in the subgroup of patients with risk factors (when stratified results were reported), the incidence of moderate to severe PEP, adverse events related to NSAID administration, mortality, and length of hospital stay.

### Quality assessment of the studies

Two independent reviewers assessed the quality of trials with the Jadad scale (AI & IP). In the case of one article published in Hungarian [11], we contacted the authors who helped us to assess the Jadad scale. Disagreements were discussed by the reviewers and resolved through consensus. According to this scale, low-quality studies had a score of ≤2 and high-quality studies had a score of ≥3 [29].

### Statistical analysis

Differences observed between the groups were expressed as risk ratios (RR) with their 95% confidence intervals (CI). In the case of primary and secondary endpoints, numbers needed to treat (NNT) were also calculated. Heterogeneity was measured using the I^2^ test and was considered significant when the I^2^ value was above 50% [30], [31]. A fixed-effects model weighted by the Mantel-Haenszel method was used for pooling the RR’s because of the low heterogeneity between studies. Funnel plot was used to estimate the risk of publication bias. All calculations were performed using the freeware Review Manager 5.1 (Cochrane Foundation, McMaster University, Ontario, Canada) [32].

## Results

Original searches retrieved more than 1000 articles. After review of the abstracts, 12 studies met the inclusion criteria. After careful evaluation, nine were included in the systematic review and the meta-analysis (Figure 1).

### Studies excluded

Three studies were finally excluded from the meta-analysis: 1) Montaño Loza et al [33] reported the preliminary results of another study which was already included; 2) Katsinelos et al [34] administered somatostatin along with NSAIDs and found a significantly lower incidence of PEP in this treatment group (4.7% vs 10.4%, p = 0.015); 3) Bhatia et al [35] did not find statistically significant differences when comparing valdecoxib iv vs. placebo (9.9% vs 10.3%, p = 0.99). This study was excluded because valdecoxib is a selective COX2 inhibitor. The effect of coxibs in reducing PLA2 activity is unknown, and therefore they may not be as effective as conventional NSAID for PEP prevention.

### Studies included

Nine studies, all published as full text articles, were included [10]–[18]. The characteristics and quality of the studies are shown in table 1. Six studies were scored as high-quality (Jadad score 5) [10]–[13], [15], [18] and three as low-quality (Jadad score 2) [14], [16], [17].

**Table 1:** Basic characteristics of included studies in the meta-analysis.

Author, year, country	N	Inclusion criteria	Does the study include patients with a pancreatic stent for PEP prophylaxis?	Intervention	Definition of PEP	Jadad score
Murray, 2003, Scotland [15]	220	High risk patients (Pancreatography or cholangiogaphy with SOH)	Yes^a^^,^ b	100 mg rectal diclofenac in recovery area	Amylase >x4 ULN and epigastric pain, back pain and abdominal rebound tenderness	5
Sotoudehmanesh, 2007, Iran [18]	442	Unselected patients (ERCP, all-comers)	No	100 mg rectal indomethacin immediately prior to ERCP	Amylase >x3 ULN and epigastric or back pain and epigastric tenderness	5
Khoshbaten, 2008, Iran [13]	200	High risk patients (Pancreatography ± cholangiography)	Yes^a^^,^ b	100 mg rectal diclofenac on arrival in recovery area	Amylase >x4 ULN and epigastric and back pain and epigastric rebound tenderness	5
Montaño Loza, 2007, Mexico [14]	150	Unselected patients (ERCP, suspected bile duct obstruction)	No	100 mg rectal indomethacin immediately prior to ERCP	Amylase >x3 ULN + sharp pain radiating to back + nausea or vomiting	2
Cheon, 2007, USA [10]	207	Unselected patients (ERCP, all-comers)	Yes^b^	50 mg diclofenac before and after ERCP by mouth	Amylase >x3 ULN 18h after ERCp + abdominal pain that prolonged hospital stay	5
Senol, 2009, Turkey [17]	80	Unselected patients (ERCP, cholestasis)	No	75 mg diclofenac im and i.v. isotonic after ERCP	Amylase >x3 ULN + epigastric pain or back pain + epigastric tenderness	2
Otsuka, 2012, Japan [16]	104	Unselected patients (ERCP, all-comers)	No	50 mg rectal diclofenac 30 mins before ERCP	Amylase >x3 ULN + abdominal pain within 24h after ERCP	2
Elmunzer, 2012, USA [12]	602	High risk patients (1 major or 2 minor previously defined risk factors)	Yes^b^	100 mg rectal indomethacin after ERCP	Amylase >x3 ULN +upper abdominal pain 24 h after ERCP + hospitalization for ≥ 2 nights	5
Döbrönte, 2012, Hungary [11]	228	Unselected patients (ERCP, all-comers)	No	100 mg rectal indomethacin 10 mins before ERCP	Amylase >x3 ULN, abdominal pancreatic pain within 24h after ERCP and extension of hospitalization	5

a A pancreatic stent was placed only in 25 and 5 patients in these studies.

b Not statistically significant differences between placebo and NSAIDs group.

*ERCP:* endoscopic retrograde cholangiopancreatography; *NSAIDs:* non-steroidal anti-inflammatory drugs; *PEP:* post-ERCP pancreatitis; *SOH:* sphincter of Oddi hypertension; *ULN:* upper limit of normal.

According to the inclusion criteria (table 1), three studies included only high risk patients for PEP: Murray et al [15] included only patients with pancreatography or cholangiography and manometrically documented SOH; Khoshbaten et al [13] included patients with pancreatography with or without cholangiography; Elmunzer et al [12] included selected high risk patients having at least one major or two minor criteria which had been previously defined. The remaining six studies [10], [11], [14], [16]–[18] included unselected patients.

Risk factors for PEP were similar for both the treatment and the placebo arms in all studies. Only one study [16] reported a statistically significant difference in sex distribution: 31 women were included in the diclofenac group *vs.* 20 in the placebo group; p value was 0.019.

Pancreatic stent for PEP prophylaxis was placed in selected patients in four studies [10], [12], [13], [15]. The rate of stent placement was similar in the NSAID and the placebo groups. Five studies reported that they did not use pancreatic stent for PEP prophylaxis [11], [14], [16]–[18].

Main exclusion criteria were the same for all the studies: NSAID use immediately before inclusion in the study, the presence of active pancreatitis before ERCP and the existence of contraindications for NSAID administration.

Four studies [10], [12], [16], [18] graded the severity of post-ERCP pancreatitis according to the criteria proposed by Cotton et al. [24] and accepted in the current guidelines [4]: mild PEP was defined as the need for hospital admission or prolongation of planned admission up to three days; moderate PEP is defined by the need for hospitalization lasting 4 – 10 days, and severe PEP by hospitalization for more than 10 days, or necrosis or pseudocyst, or need for percutaneous drainage or surgical intervention. Montaño Loza et al [14] graded the severity of pancreatitis according to Ranson’s criteria, while Murray et al [15] graded it according to CT findings.

Length of hospital stay was reported in different ways. Murray et al [15] and Elmunzer et al [12] reported the median length of hospital stay only for the patients who developed PEP in each arm. Cheon et al [10] reported the median length of hospital stay for all the patients included in each arm, not those with pancreatitis. Finally, in four additional studies [10], [12], [16], [18] the number of patients with pancreatitis needing hospitalization for more than three days could be extracted because they reported the number of moderate and severe pancreatitis, which had previously been defined according to hospital stay.

### Post-ERCP pancreatitis incidence

**Figure 2:**
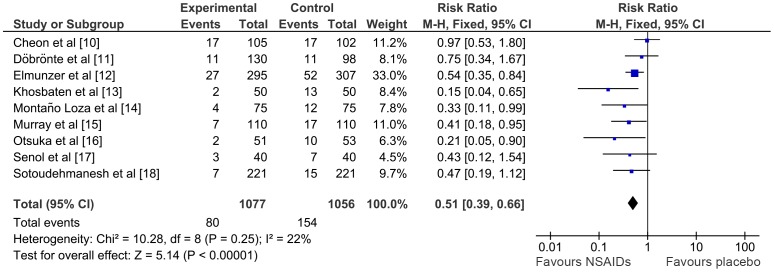
Meta-analysis comparing NSAIDs vs. placebo in reducing the number of patients with PEP. *CI,* confidence interval; *M-H*, Mantel-Haenszel; *NSAIDs*, non-steroidal anti-inflammatory drugs.

**Figure 3:**
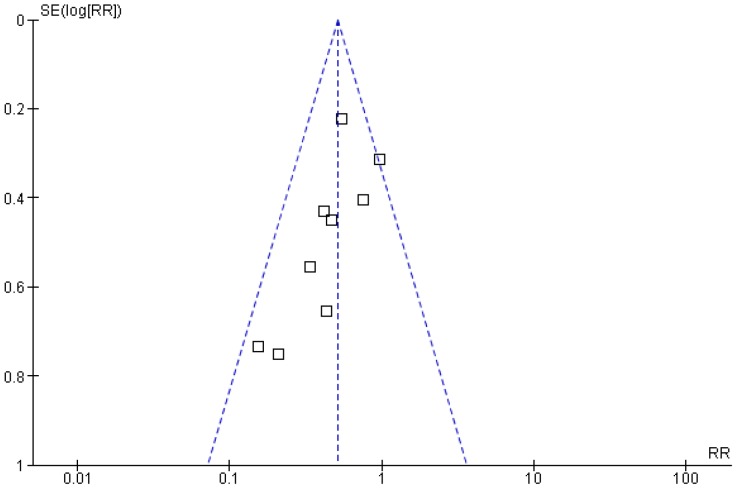
Funnel plot of the meta-analysis.

Nine studies including 2133 patients reported the number of cases of pancreatitis in the NSAID and the placebo arms. Heterogeneity between these studies was low (I^2^ = 22%). PEP occurred in 80 out of 1077 patients (7.4%) in the NSAID group vs. 154 out of 1056 patients (14.6%) in the placebo group (RR 0.51; 95% CI 0.39–0.66; p< 0.00001) (Figure 2). NNT was 14. The funnel plot showed a visual trend suggesting that small studies reported the highest risk reductions, inducing a publication bias (Figure 3). However, even though the reduction was lower, large studies also found a significant reduction in the risk of pancreatitis.

### Risk of moderate to severe pancreatitis, adverse events and mortality and length of hospital stay

**Figure 4:**
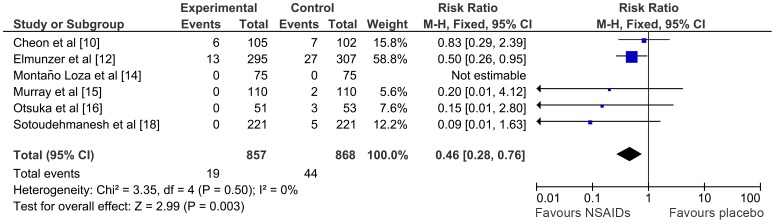
Meta-analysis comparing NSAIDs vs. placebo in reducing the number of moderate to severe pancreatitis. *CI,* confidence interval; *M-H*, Mantel-Haenszel; *NSAIDs*, non-steroidal anti-inflammatory drugs.

Six studies [10], [12], [14]–[16], [18] reported pancreatitis severity. NSAIDs were effective in reducing the incidence of moderate to severe pancreatitis compared with placebo (RR 0.46; 95% CI 0.28–0.76, p = 0.003; I^2^: 0%) (Figure 4). NNT was 33.

Regarding adverse events, Elmunzer et al [12] reported four cases of gastrointestinal bleeding in the NSAID group compared to seven in the placebo group. Cheon et al. [10] reported six cases of post endoscopic sphincterotomy (ES) bleeding during the procedure in the placebo group and eight cases in the diclofenac group (*P* = 0.921). Two cases (one in each group) required epinephrine injection. No cases of delayed bleeding were observed. Senol et al [17] also reported two cases of post ES bleeding in the control group and one case in the diclofenac group: all of them were self-limited and stopped during endoscopy, without intervention. The remaining studies did not report any NSAID-related adverse event. No deaths occurred in any study.

A reduced median length hospital stay in patients with pancreatitis was reported by Murray et al [15] (three days in the seven patients with pancreatitis in the experimental group *vs.* five days in the 17 patients with pancreatitis in the control group, P not reported) and by Elmunzer et al [12] (3.5 days in the 27 patients with pancreatitis in the indomethacin group vs. four days in the 52 patients with pancreatitis in the control group, P<0.001). Cheon et al [10] reported a non-significant reduction in the median length of hospital stay for all patients in the diclofenac arm. Hospital stay was 0.73 days in the 105 patients in the diclofenac group vs. 1.3 days in the 102 patients in the placebo group. Finally, the number of patients with pancreatitis requiring a hospital stay of more than three days was reported in four studies [10], [12], [16], [18]. Meta-analyses considering the number of patients with hospital stay of more than three days also showed a trend towards a reduced hospital stay in the experimental group, but the differences were not significant (RR 0.80; 95% CI 0.53–1.21, p = 0.3; I^2^: 0%).

### Type of NSAIDs, route and time of administration and risk factors for PEP

**Figure 5:**
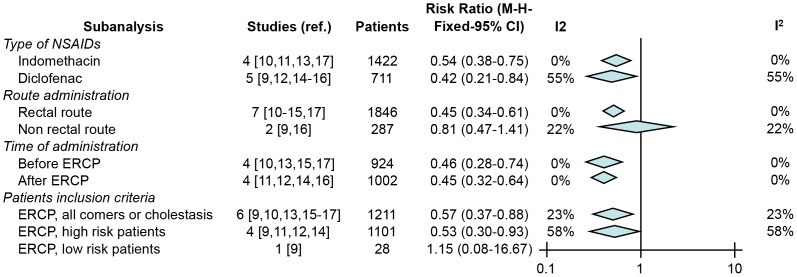
Summary of subanalyses comparing NSAIDs vs. placebo in reducing the number of patients with PEP according to: type of NSAID, route and time of administration and patients inclusion criteria.

The results of the different sub-analyses are summarized in Figure 5. Both indomethacin and diclofenac induced statistically significant reductions in the risk of pancreatitis (RR 0.54; 95% CI 0.38–0.75, p = 0.0002; I^2^: 0% and RR 0.42; 95% CI 0.21–0.84, p = 0.01; I^2^: 54%). Rectal administration was the only effective route (RR 0.45; 95% CI 0.34–0.61, p<0.0001; I^2^: 0%), while other routes – oral in one study and intramuscular in another – showed a non-significant benefit (RR 0.81; 95% CI 0.47–1.41, p = 0.46; I^2^: 22%). NSAIDs were effective when administered either before or after the ERCP (RR 0.46; 95% CI 0.28–0.74, p = 0.001; I^2^: 0% and RR 0.45; 95% CI 0.32–0.64, p<0.0001; I^2^: 0%), and in both high risk patients and non-selected patients (RR 0.53; 95% CI 0.30–0.93, p = 0.03; I^2^: 58% and RR 0.57; 95% CI 0.37–0.88; I^2^: 23%, p = 0.01).

Few studies reported the incidence of PEP according to each risk factor. In order to avoid misleading results, studies that administered NSAIDs through non-rectal routes (which did not prove effective) were not considered. Rectal administration of NSAIDs was effective in young people and females and did not depend on whether a sphincterotomy or pancreatography was performed. NSAIDs were also equally effective in patients with and without suspected or confirmed sphincter of Oddi hypertension (table 2). NSAIDs showed a benefit regardless of the placement or non-placement of a prophylactic stent. By contrast, they were not effective in old patients and in men. The data available for other risk factors were insufficient to perform a meta-analysis.

**Table 2:** Subanalysis: rectal NSAIDs *vs.* placebo in reducing the number of pancreatitis in subgroup of patients with a risk factor.

	Subanalysis	Studies [ref.]	Patients	Risk Ratio (M-H-Fixed-95% CI)	I^2^
Age					
	Young patients	2 [12], [18]	527	0.50 (0.30–0.83)	0%
	Old patients^a^	2 [12], [18]	570	0.57 (0.32, 1.05)	0%
Sex					
	Females	2 [12], [18]	513	0.54 (0.34–0.85)	0%
	Males	2 [12], [18]	327	0.54 (0.23, 1.24)	8%
Sphincterotomy					
	Yes	4 [12], [15], [16], [18]	786	0.53 (0.35–0.83)	0%
	No	4 [12], [15], [16], [18]	572	0.41 (0.23–0.72)	0%
Suspected or confirmed SOH					
	Yes	2 [12], [15]	662	0.50 (0.31–0.83)	10%
	No	2 [12], [15]	274	0.36 (0.17–0.77)	0%
Pancreatic duct injection					
	Yes	3 [13], [15], [18]	454	0.29 (0.13–0.63)	0%
	No	1 [18]	348	0.62 (0.21–1.80)	-
Prophylactic pancreatic stent^b^					
	Yes	1 [12]	496	0.61 (0.38–0.98)	-
	No	5 [11], [12], [16]–[18]	960	0.45 (0.28–0.70)	0%

*SOH:* sphincter of Oddi hypertension; *ERCP:* endoscopic retrograde cholangiopancreatography; *NSAIDs:* non-steroidal anti-inflammatory drugs.

a The two different studies used different cut-off values to separate young and old patients, one using 45 years and the other 60 years.

b Prophylactic measure, not risk factor.

## Discussion

The present study showed that rectal administration of indomethacin or diclofenac, before or after ERCP, is an efficacious and safe measure for reducing the incidence and the severity of post-ERCP pancreatitis. According to the current data, either diclofenac or indomethacin can be used interchangeably. A single rectal dose administered either before or immediately after ERCP seems the most suitable schedule. The sub-analysis showed that rectal NSAIDs were beneficial in both high-risk patients (including those with sphincterotomy, pancreatography and suspected or confirmed SOH) and in unselected patients. Finally, rectal NSAIDs appear to be effective whether or not a prophylactic pancreatic stent was inserted.

Two previous meta-analyses with few studies conclude that NSAIDs are effective in preventing PEP [20], [21]. The recent meta-analysis published by Ding et al [22] includes additional studies, but one was reported only in abstract form and is as yet unpublished as a full text, and another evaluated the use of the Cox2 inhibitor valdecoxib. The authors conclude that NSAIDs reduce the incidence and severity of PEP. However, our meta-analysis, including an additional randomized controlled trial [11], determines that the evidence is conclusive only for rectal NSAIDs. In addition, our study highlights some clinically relevant issues: rectal NSAIDs may be administered either before or after ERCP, both indomethacin and diclofenac seem effective, and NSAIDs are effective in both selected and unselected patients. These data agree with previous sub-analyses published by Zheng et al [36]. Although a trend towards reducing length of hospital stay was observed, few data were reported in the studies and so this cannot be confirmed.

A first limitation of the meta-analysis is a possible overestimation of the risk reduction because of the high prevalence of PEP in the control group of the included studies (7–26%). This high incidence contrasts with the rates reported in previous series that range from 1% to 10% [25], [28], [37]. A reasonable explanation for this high complication rate is that many studies include high-risk patients. Another possible limitation is that a proportion of the studies [14], [16], [17] were rated as low-quality (Jadad = 2) [38]. However, the risk reduction observed when low-quality studies were excluded was very similar to those of the main analysis (RR 0.55; 95% CI 0.42–0.73, I^2^: 33%, data not shown). In addition, severity of pancreatitis was assessed by Ranson’s criteria in one study [14] and by CT findings in another [15], rather than using the currently accepted consensus definition proposed by Cotton [4]. However the risk reduction of moderate and severe pancreatitis when these studies were excluded was also very similar to the main group analysis (RR 0.47; 95% CI 0.28–0.80, data not shown). Finally, some of the sub-analyses should be interpreted with great caution because they include few studies and a low number of patients. The reduced power of some of the comparisons may explain the non-significant results in males and older age.

Our findings suggest that rectal NSAIDs are also effective when a pancreatic stent is placed. Elmunzer et al. [39] concludes in a post hoc analysis that rectal indomethacin could replace the placement of pancreatic stents. However, no RCTs comparing rectal NSAIDs alone vs. NSAIDs plus pancreatic stent placement have been carried out to date. Combinations of these agents, which act on different steps in the pathogenesis of post-ERCP pancreatitis, may reduce PEP even more in selected high risk patients, but adequately performed RCTs are needed to confirm this point.

In conclusion, there is evidence supporting the rectal administration of indomethacin or diclofenac, either before or immediately after ERCP. There is no evidence to recommend oral or parenteral administration. Rectal NSAID seem to be safe and effective in reducing the incidence and the severity of post-ERCP pancreatitis, both in high risk patients and unselected patients.

## Supporting Information

Appendix S1 **Search strategy used in Scopus, Pubmed, ISI Web of Knowledge and the Cochrane Library.**
(DOC)

Checklist S1 **PRISMA 2009 checklist.**
(DOC)

## References

[1] Freeman ML, Guda NM (2004) Prevention of post-ERCP pancreatitis: a comprehensive review. Gastrointest Endosc 59: 845–64. 15173799 10.1016/s0016-5107(04)00353-0

[2] Masci E, Toti G, Mariani A, Curioni S, Lomazzi A, et al (2001) Complications of diagnostic and therapeutic ERCP: a prospective multicenter study. Am J Gastroenterol 96: 417–23. 11232684 10.1111/j.1572-0241.2001.03594.x

[3] Cheng CL, Sherman S, Watkins JL, Barnett J, Freeman M, et al (2006) Risk factors for post-ERCP pancreatitis: a prospective multicenter study. Am J Gastroenterol 101: 139–47. 16405547 10.1111/j.1572-0241.2006.00380.x

[4] Dumonceau JM, Andriulli A, Deviere J, Mariani A, Rigaux J, et al (2010) European Society of Gastrointestinal Endoscopy (ESGE) Guideline: prophylaxis of post-ERCP pancreatitis. Endoscopy 42: 503–15. 20506068 10.1055/s-0029-1244208

[5] Gross V, Leser HG, Heinisch A, Scholmerich J (1993) Inflammatory mediators and cytokines—new aspects of the pathophysiology and assessment of severity of acute pancreatitis? Hepatogastroenterol 40: 522–30. 7509768

[6] Bhatia M, Brady M, Shokuhi S, Christmas S, Neoptolemos JP, et al (2000) Inflammatory mediators in acute pancreatitis. J Pathol 190: 117–25. 10657008 10.1002/(SICI)1096-9896(200002)190:2<117::AID-PATH494>3.0.CO;2-K

[7] Karne S, Gorelick FS (1999) Etiopathogenesis of acute pancreatitis. Surg Clin North Am 79: 699–710. 10470320 10.1016/s0039-6109(05)70036-0

[8] Makela A, Kuusi T, Schroder T (1997) Inhibition of serum phospholipase-A2 in acute pancreatitis by pharmacological agents in vitro. Scand J Clin Lab Invest 57: 401–7. 9279965 10.3109/00365519709084587

[9] Kozarek RA (1990) Pancreatic stents can induce ductal changes consistent with chronic pancreatitis. Gastrointest Endosc 36: 93–5. 2335298 10.1016/s0016-5107(90)70958-3

[10] Cheon YK, Cho KB, Watkins JL, McHenry L, Fogel EL, et al (2007) Efficacy of diclofenac in the prevention of post-ERCP pancreatitis in predominantly high-risk patients: a randomized double-blind prospective trial. Gastrointest Endosc 66: 1126–32. 18061712 10.1016/j.gie.2007.04.012

[11] Dobronte Z, Toldy E, Mark L, Sarang K, Lakner L (2012) Effects of rectal indomethacin in the prevention of post-ERCP acute pancreatitis. Orvosi hetilap 153: 990–6. 22714033 10.1556/OH.2012.29403

[12] Elmunzer BJ, Scheiman JM, Lehman GA, Chak A, Mosler P, et al (2012) A randomized trial of rectal indomethacin to prevent post-ERCP pancreatitis. N Engl J Med 366: 1414–22. 22494121 10.1056/NEJMoa1111103 PMC3339271

[13] Khoshbaten M, Khorram H, Madad L, Ehsani Ardakani MJ, Farzin H, et al (2008) Role of diclofenac in reducing post-endoscopic retrograde cholangiopancreatography pancreatitis. J Gastroenterol Hepatol 23: 11–6. 10.1111/j.1440-1746.2007.05096.x 17683501

[14] Montano Loza A, Rodriguez Lomeli X, Garcia Correa JE, Davalos Cobian C, Cervantes Guevara G, et al (2007) Effect of the administration of rectal indomethacin on amylase serum levels after endoscopic retrograde cholangiopancreatography, and its impact on the development of secondary pancreatitis episodes. Rev Esp Enferm Dig 99: 330–6. 17883296 10.4321/s1130-01082007000600005

[15] Murray B, Carter R, Imrie C, Evans S, O'Suilleabhain C (2003) Diclofenac reduces the incidence of acute pancreatitis after endoscopic retrograde cholangiopancreatography. Gastroenterology 124: 1786–91. 12806612 10.1016/s0016-5085(03)00384-6

[16] Otsuka T, Kawazoe S, Nakashita S, Kamachi S, Oeda S, et al (2012) Low-dose rectal diclofenac for prevention of post-endoscopic retrograde cholangiopancreatography pancreatitis: a randomized controlled trial. J Gastroenterol 47: 912–7. 22350703 10.1007/s00535-012-0554-7

[17] Senol A, Saritas U, Demirkan H (2009) Efficacy of intramuscular diclofenac and fluid replacement in prevention of post-ERCP pancreatitis. World J Gastroenterol 15: 3999–4004. 19705494 10.3748/wjg.15.3999 PMC2731949

[18] Sotoudehmanesh R, Khatibian M, Kolahdoozan S, Ainechi S, Malboosbaf R, et al (2007) Indomethacin may reduce the incidence and severity of acute pancreatitis after ERCP. Am J Gastroenterol 102: 978–83. 17355281 10.1111/j.1572-0241.2007.01165.x

[19] Dumonceau JM, Rigaux J, Kahaleh M, Gomez CM, Vandermeeren A, et al (2010) Prophylaxis of post-ERCP pancreatitis: a practice survey. Gastrointest Endosc 71: 934–9. 20226455 10.1016/j.gie.2009.10.055

[20] Elmunzer BJ, Waljee AK, Elta GH, Taylor JR, Fehmi SM, et al (2008) A meta-analysis of rectal NSAIDs in the prevention of post-ERCP pancreatitis. Gut 57: 1262–7. 18375470 10.1136/gut.2007.140756

[21] Dai HF, Wang XW, Zhao K (2009) Role of nonsteroidal anti-inflammatory drugs in the prevention of post-ERCP pancreatitis: a meta-analysis. Hepatobiliary Pancreat Dis Int 8: 11–6. 19208508

[22] Ding X, Chen M, Huang S, Zhang S, Zou X (2012) Nonsteroidal anti-inflammatory drugs for prevention of post-ERCP pancreatitis: a meta-analysis. Gastrointest Endosc 76: 1152–9. 23164513 10.1016/j.gie.2012.08.021

[23] Liberati A, Altman DG, Tetzlaff J, Mulrow C, Gotzsche PC, et al (2009) The PRISMA statement for reporting systematic reviews and meta-analyses of studies that evaluate health care interventions: explanation and elaboration. PLoS Medicine 6: e1000100. 19621070 10.1371/journal.pmed.1000100 PMC2707010

[24] Cotton PB, Lehman G, Vennes J, Geenen JE, Russell RC, et al (1991) Endoscopic sphincterotomy complications and their management: an attempt at consensus. Gastrointest Endosc 37: 383–93. 2070995 10.1016/s0016-5107(91)70740-2

[25] Freeman ML, Nelson DB, Sherman S, Haber GB, Herman ME, et al (1996) Complications of endoscopic biliary sphincterotomy. N Engl J Med 335: 909–18. 8782497 10.1056/NEJM199609263351301

[26] Choi CW, Kang DH, Kim GH, Eum JS, Lee SM, et al (2009) Nafamostat mesylate in the prevention of post-ERCP pancreatitis and risk factors for post-ERCP pancreatitis. Gastrointest Endosc 69: 11–8. 10.1016/j.gie.2008.10.046 19327467

[27] Lee KT, Lee DH, Yoo BM (2008) The prophylactic effect of somatostatin on post-therapeutic endoscopic retrograde cholangiopancreatography pancreatitis: a randomized, multicenter controlled trial. Pancreas 37: 445–8. 18953260 10.1097/MPA.0b013e3181733721

[28] Masci E, Mariani A, Curioni S, Testoni PA (2003) Risk factors for pancreatitis following endoscopic retrograde cholangiopancreatography: a meta-analysis. Endoscopy 35: 830–4. 14551860 10.1055/s-2003-42614

[29] Kjaergard LL, Villumsen J, Gluud C (2001) Reported methodologic quality and discrepancies between large and small randomized trials in meta-analyses. Ann Intern Med 135: 982–9. 11730399 10.7326/0003-4819-135-11-200112040-00010

[30] Higgins JP, Thompson SG (2002) Quantifying heterogeneity in a meta-analysis. Stat Med 21: 1539–58. 12111919 10.1002/sim.1186

[31] Higgins JP, Thompson SG, Deeks JJ, Altman DG (2003) Measuring inconsistency in meta-analyses. BMJ 327: 557–60. 12958120 10.1136/bmj.327.7414.557 PMC192859

[32] Review Manager (RevMan) (2011) Version 5.1 ed: Copenhagen: The Nordic Cochrane Centre, The Cochrane Collaboration.

[33] Montano Loza A, Garcia Correa J, Gonzalez Ojeda A, Fuentes Orozco C, Davalos Cobian C, et al (2006) Prevention of hyperamilasemia and pancreatitis after endoscopic retrograde cholangiopancreatography with rectal administration of indomethacin. Rev Gastroenterol Mex 71: 262–8. 17140047

[34] Katsinelos P, Fasoulas K, Paroutoglou G, Chatzimavroudis G, Beltsis A, et al (2012) Combination of diclofenac plus somatostatin in the prevention of post-ERCP pancreatitis: a randomized, double-blind, placebo-controlled trial. Endoscopy 44: 53–9. 22198776 10.1055/s-0031-1291440

[35] Bhatia V, Ahuja V, Acharya SK, Garg PK (2011) A randomized controlled trial of valdecoxib and glyceryl trinitrate for the prevention of post-ERCP pancreatitis. J Clin Gastroenterol 45: 170–6. 20717044 10.1097/MCG.0b013e3181eb600e

[36] Zheng MH, Xia HH, Chen YP (2008) Rectal administration of NSAIDs in the prevention of post-ERCP pancreatitis: a complementary meta-analysis. Gut 57: 1632–3. 18941015

[37] Cheon YK, Cho KB, Watkins JL, McHenry L, Fogel EL, et al (2007) Frequency and severity of post-ERCP pancreatitis correlated with extent of pancreatic ductal opacification. Gastrointest Endosc 65: 385–93. 17321236 10.1016/j.gie.2006.10.021

[38] Moher D, Pham B, Jones A, Cook DJ, Jadad AR, et al (1998) Does quality of reports of randomised trials affect estimates of intervention efficacy reported in meta-analyses? Lancet 352: 609–13. 9746022 10.1016/S0140-6736(98)01085-X

[39] Elmunzer BJ, Higgins PDR, Saini SD, Scheiman JM, Parker RA, et al (2013) Does rectal indomethacin eliminate the need for prophylactic pancreatic stent placement in patients undergoing high-risk ercp post hoc efficacy and cost-benefit analyses using prospective clinical trial data. Am J Gastroenterol 108: 410–415. 23295278 10.1038/ajg.2012.442 PMC3947644

