# Supplementary material for: How and When Should NSAIDs Be Used for Preventing Post-ERCP Pancreatitis? A Systematic Review and Meta-Analysis
Source: PLoS One. 2014 Mar 27;9(3):e92922. doi: 10.1371/journal.pone.0092922 (PMC3968039; doi:10.1371/journal.pone.0092922)
Supplement: Appendix S1 — Search strategy used in Scopus, Pubmed, ISI Web of Knowledge and the Cochrane Library. (DOC) [file pone.0092922.s001.doc]

Appendix S1. Search strategy used in Scopus, Pubmed, ISI Web of Knowledge and the Cochrane Library

(NSAIDs OR indomethacin OR diclofenac OR ibuprof* OR metamiz* OR pyrazolon* OR dypiron* OR aspirin* OR ketoprofen* OR naproxen OR ketorolac OR piroxicam OR meloxicam) AND pancreatitis
